# Data on food insufficiency status in South Africa: Insight from the South Africa General Household Survey

**DOI:** 10.1016/j.dib.2019.103730

**Published:** 2019-03-07

**Authors:** Abiodun Olusola Omotayo, Adebayo Isaiah Ogunniyi, Adeyemi Oladapo Aremu

**Affiliations:** a Food Security and Safety Niche Area, Faculty of Natural and Agricultural Sciences, North West University, Private Mail Bag X2046, Mmabatho, 2790, North West Province, South Africa; b International Food Policy Research Institute (IFPRI), Abuja, Nigeria; c Indigenous Knowledge Systems (IKS) Centre, Faculty of Natural and Agricultural Sciences, North West University, Private Mail Bag X2046, Mmabatho, 2790, North West Province, South Africa

**Keywords:** Food security, Children, Adult, Data, Sustainable goal

## Abstract

Food insecurity or insufficiency, among other factors, is triggered by structural inequalities. Food insecurity is an inflexible problematic situation in South Africa. The country has a custom of evidence-based decision making, stocked in the findings of generalized national household surveys. Conversely, the deep insights from the heterogeneity of the sub-national analysis remain a principally unexploited means of understanding of the contextual experience of food insecurity or insufficiency in South Africa. The data present the food insufficiency status with special focus on adult and children. The data also reveal the adult and children food insufficiency status across the provinces in South Africa. The data contains socioeconomic and demographic characteristics as well the living condition and food security status of the households.

**Table:** Specifications table

Subject area	Agricultural Economics, Economics
More specific subject area	Food security and livelihood outcomes
Type of data	Table, Dta. File, text file, Figure
How data was acquired	Household survey
Data format	Raw, analyzed, descriptive and statistical data
Experimental factors.	Samples consist of all private households in all the nine provinces of South Africa and residents in workers' hostels.
Experimental features	There was no experimental component in the dataset used
Data source location	9 provinces in South Africa; Western Cape, Eastern Cape, Northern Cape, North West, Free State, Kwazulu Natal, Gauteng, Limpopo and Mpumalanga
Data accessibility	The datasets explored and analyzed are available at http://microdata.worldbank.org/index.php/catalog/2559
Related research article	None

**Table:** 

**Value of the data** • These data present information on the socioeconomic and demographic characteristics of household as it relates with food (in) security of the household members. This dataset will provide valuable information that may be functional at different levels for both government organizations (GOs) and non-government organizations (NGOs) in order to formulate appropriate policy and intervention strategy for the improvement of food for poor households in South Africa. • This data allows other researchers to extend the statistical analyses in various dimension of measuring livelihood outcomes in South Africa.

## 1 Data

Data was made available with a well-structured household questionnaire with a unit of analysis captured at households and individuals level. A questionnaire was administered to a household to elicit information on household members. The survey covers all legally recognized household members (usual residents) of households in the nine provinces (Eastern Cape, Free State, Gauteng, KwaZulu-Natal, Limpopo, Mpumalanga, the Northern Cape, North West, and the Western Cape) of South Africa. The survey does not cover collective living quarters such as student hostels, old-age homes, hospitals, prisons, and military barracks but specifically on households. The General Household Survey (GHS) collects data on education, health and social development, housing, access to services and facilities, food security, and agriculture.

The data in Table 1 show the socioeconomics and demographics characteristics of the household heads sampled in South Africa. The mean age was found to be 47.8 years (approximately 48 years) and more than half were male. The representation in this data is typical of Sub Saharan African countries [1], [2]. The highest (52.1%) source of income is through salaries or wages or commission while just 1.1% earn income through agricultural sales. The data show that over 80% of the respondents are South African/Black race while the Indian/Asia race have the least (Table 1).

**Table 1:** Summary statistics of some selected variables.

Variable	Observation	Mean	Std. Dev.
Age of the household head	21,218	47.822	15.833
Male-Headed households	21,218	0.575	0.494
Involve in agricultural job	21,218	0.173	0.379
Income per month	21,218	9628.212	17,714.46
**Income sources**			
Salaries/wages/commission	21,218	0.521	0.499
Income from a business	21,218	0.073	0.261
Remittances	21,218	0.082	0.274
Pensions	21,218	0.020	0.140
Grants (include old age grant	21,218	0.244	0.429
Sales of farming products and services	21,218	0.001	0.034
Other income sources e.g.	21,218	0.011	0.106
No income	21,218	0.008	0.092
Unspecified	21,218	0.036	0.186
**Living condition**			
Electricity access	21,218	0.931	0.253
Good walling condition	21,218	0.657	0.474
Good roofing condition	21,218	0.621	0.484
Flooring condition	21,218	0.702	0.456
Improved sanitation access	21,218	1	0
Improved water access	21,218	1	0
**Province**			
Western Cape	21,218	0.101	0.301
Eastern Cape	21,218	0.132	0.339
Northern Cape	21,218	0.0434	0.203
Free State	21,218	0.061	0.240
KwaZulu-Natal	21,218	0.160	0.366
North West	21,218	0.069	0.253
Gauteng	21,218	0.239	0.426
Mpumalanga	21,218	0.081	0.273
Limpopo	21,218	0.109	0.312
**Race**			
African/Black	21,218	0.820	0.383
Colored	21,218	0.080	0.271
Indian/Asian	21,218	0.020	0.141
White	21,218	0.078	0.269

Source: Authors compilation, 2018.

**Fig. 1:**
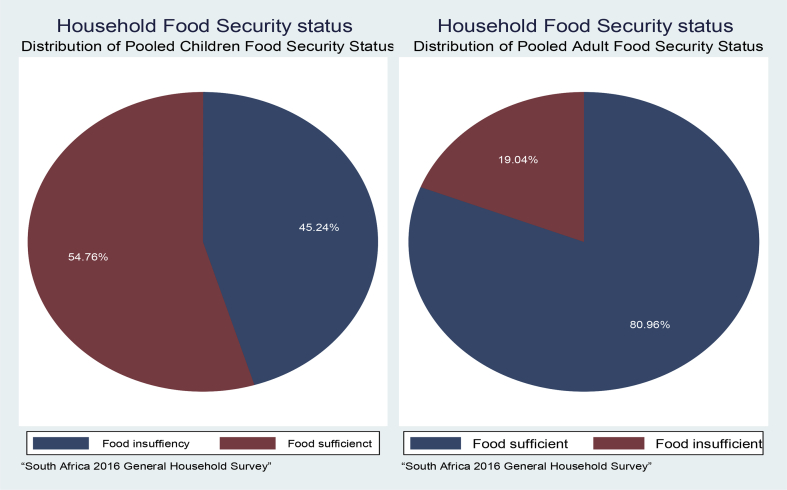
Food security status in among children and adults in South Africa. Source: Authors computation, 2018.

In Fig. 1, using Foster–Greer–Thorbecke index (FGT Index) as well as descriptive analysis, the data show that children experienced food insufficiency more than adults in South Africa. The data reveal that over 40 percent of the children are living in household experiencing food insufficiency.

**Fig. 2:**
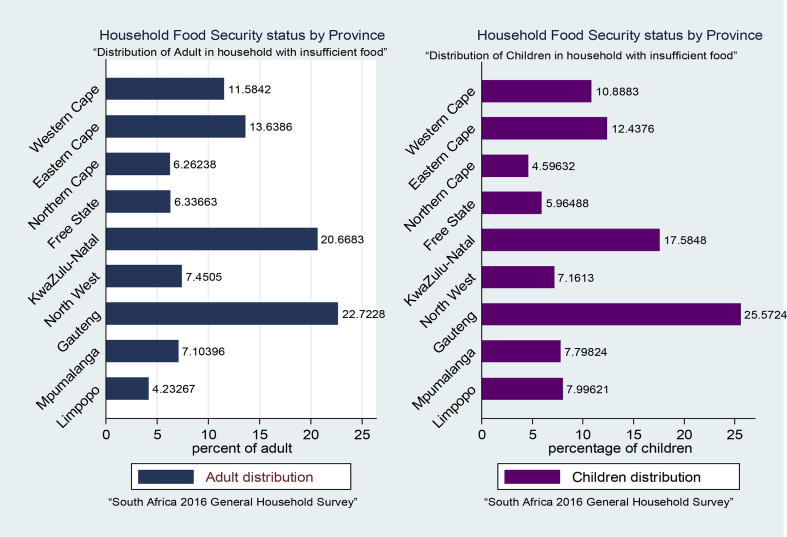
Disaggregated food security status across the nine provinces in South Africa. Source: Authors computation, 2018.

In the same vein, the data in Fig. 2 show the disaggregation of food security status across the 9 provinces in South Africa with special focus on children and adult. The data show that both for children and adult in, Guateng and KwaZulu-Natal experienced highest level of food insufficiency in South Africa. The data show that 22.72% and 20.66% of adult and 17.58% and 25.57% children are food insufficient in Guateng and KwaZulu-Natal province, respectively. The dataset also revealed that food insufficiency is lowest for both children (4.59) and adult (6.26) in Northern Cape Province.

## 2 Experimental design, materials and methods

The dataset employed is the General Household Survey (GHS), 2016. The dataset was compiled based on stratified two-stage design, and a total of rural and urban 21,218 households were interviewed containing 72,604 respondents. The dataset were coded in SPSS software 22 version which the descriptive part of the research such as mean, frequency, standard deviation were carried out. In addition, the inferencial statistics were carried out on STATA package 13 using the FGT index to classify the respondents into food secured or otherwise. The dataset was robust and representative enough to generalize on the household food sufficiency status of South Africa.

Acknowledgements
