# Supplementary material for: Data on food insufficiency status in South Africa: Insight from the South Africa General Household Survey
Source: Data Brief. 2019 Mar 7;23:103730. doi: 10.1016/j.dib.2019.103730 (PMC6660462; doi:10.1016/j.dib.2019.103730)
Supplement: Supplementary file 1 — Multimedia component 1 [file mmc1.docx]

Food Security Niche Area, North-West University,

Mafikeng Campus, Mmabatho, 2735,

Republic of South Africa,

2nd January, 2018.

The Editor,

Datainbrief,

Dear Sir,

**CONFLICT OF INTEREST FORM**

I hereby write to submit a manuscript for publication in your highly esteemed journal. **Data on Food Insufficiency in South Africa; Insight from South Africa General Household Survey**. The manuscript contains the report of an original data related scientific research work and it has not been published previously and is not currently submitted for review in any other journal. There is also no conflict of interest among the authors of this work. I would be grateful if the manuscript is considered suitable for publication in your journal.

Thank you for your anticipated cooperation.

Yours faithfully,

**

**Dr Abiodun Olusola Omotayo**
